# The Emergence of Relationship-based Cooperation

**DOI:** 10.1038/srep16447

**Published:** 2015-11-16

**Authors:** Bo Xu, Jianwei Wang

**Affiliations:** 1 School of Business Administration, Northeastern University, Shenyang, China, 110000

## Abstract

This paper investigates the emergence of relationship-based cooperation by coupling two simple mechanisms into the model: tie strength based investment preference and homophily assumption. We construct the model by categorizing game participants into four types: prosocialists (players who prefers to invest in their intimate friends), antisocialists (players who prefer to invest in strangers), egoists (players who never cooperate) and altruists (players who cooperate indifferently with anyone). We show that the relationship-based cooperation (prosocialists) is favored throughout the evolution if we assume players of the same type have stronger ties than different ones. Moreover, we discover that strengthening the internal bonds within the strategic clusters further promotes the competitiveness of prosocialists and therefore facilitates the emergence of relationship-based cooperation in our proposed scenarios. The robustness of the model is also tested under different strategy updating rules and network structures. The results show that this argument is robust against the variations of initial conditions and therefore can be considered as a fundamental theoretical framework to study relationship-based cooperation in reality.

We are living in a world where relationship-based cooperation is dominant. Parents devote efforts to kids, friends helps each other when they are in need. Despite that such cooperative behaviors usually require the sacrifice of the individual benefits, it turns out that this type of relationship-based cooperation is favored by natural selection^1^,^2^,^3^,^4^,^5^. Soccer teams with tighter bonds outperform their counterparts, firms with intimate interpersonal relationships operates more efficiently. These examples suggest that the performance of different organizations and the fitness of various species are positively associated with the strength of their internal bonds. Actually, relationships among players are strongly correlated with their extent of kinship, direct reciprocity, indirect reciprocity, group selection and network reciprocity in social networks, which are claims to be the five fundamental rules that boost cooperation in reality^6^. It is not surprising that social relationships can dramatically influence the behavioral patterns of individuals since all interactions of people are performed via social ties that connect them^7^,^8^,^9^,^10^,^11^,^12^,^13^,^14^,^15^,^16^,^17^,^18^. Haan *et al.* assigned high school students into groups comprised of classmates who either were or were not friends; they reported that contributions to a public good were higher when groups were composed of friends. More recently, Harrison *et al.* report that people tend to sacrifice more to support their close friends if they interact within the framework of donation game^19^. These findings suggest that it is not appropriate if we simply define a player in the game as either a cooperator or a defector. The decision to cooperate or defect depends on the specific opponent, more precisely, on the relationship between the two players^20^. A player can be a defector to his “nodding-friends” and simultaneously cooperate with his “close friends”^21^. Under such scenarios, it is actually the player’s behavioral preference that determines the final decisions in game interactions.

Based on the above observations, we propose an evolutionary game theoretic model to explain the emergence of relation-based cooperation in reality. In the classical evolutionary prisoner’s dilemma game (PDG) model, each player has two feasible actions: cooperation (C) or defection (D). Both players get *R* (reward) for mutual cooperation and *P* (punishment) for mutual defection. A defector exploiting a cooperator gets *T* (the temptation to defect) and the exploited cooperator gets *S* (the sucker’s payoff). *R, P, T, S* satisfies following conditions: *T* > *R* > *P* > *S and 2R* > *T* + *S*. To better illustrate the roles of diverse tie strengths in PDG, we consider an important special case called the “donation game” (DG) in this paper^22^,^23^,^24^,^25^,^26^,^27^. In a DG, each player can cooperate by providing a benefit *b* to the other player at his or her cost *c*, with 0 < *c* < *b*. Then, T = *b*, R = *b–c*, P = 0, and S = *−c*. The payoff matrix is (see table 1) (Table 1).

In this paper, we wish to extend the traditional evolutionary prisoner’s dilemma game model by introducing four types of players with different behavioral patterns: 1. Prosocialists: players who tends to cooperate more with strong tie friends; 2. Antisocialists: players who tends to cooperate more with weak tie friends; 3. Egoists: players who never cooperate with others; 4. Altruists: players who cooperate indifferently with all neighbors. Importantly, we assume that the tie strengths between players of the same type are stronger than those of different types. In social network literatures, this assumption is termed as homophily or “birds of a feather”^28^, which elaborates the fact that similar individuals tend to form strong ties.

Under such simple settings, this research constructs a theoretical framework to explore the evolutionary process of relationship-based interactions and explain the emergence of prosocial cooperation observed in social life. We reveal that the existence of heterogeneous tie strength and the resulted preferential investment preference enables prosocialists outperform other types of players throughout evolution. Moreover, strengthening such investment preference can further enhance the level of prosocial cooperation in prisoner’s dilemma games.

The remainder of this paper is organized as follows: Section 2 describes the mechanism of the relationship-based game model. Simulation results are discussed in section 3. In section 4, we summarize the results and outline some important implications of our findings.

## The Model

We consider a *L* × *L* periodic square lattice with four types of players equally distributed initially. Each player is allowed to interact with its four neighbors and self-interactions are excluded. Let *TS*_*ij*_ denotes the tie strength value between player *i* and *j*. The initial strength of each tie *TS*_*ij*_ is randomly chosen from the interval [0, 2], where we assume 1 is the boundary of strong ties and weak ties. A strong relationship has a tie strength value between^1^,^2^ and the strength of a weak relationship is between the interval [0, 1]. In each round, a randomly selected player *x* is allowed to adopt the strategy of a randomly selected friend *y* with a probability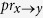 proportional to their payoff difference. It is worth mentioning that this proposed model adopts the assumption of homophily during the strategy updating process, where ties between players of the same type are assumed to be stronger than those between different types. This mechanism is realized by assigning a strong tie strength value 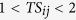 if *i* imitates *j*’s strategy in the previous round. Otherwise, if *i* and *j* have different behavioral patterns and *i* doesn’t learn from *j* after payoff comparison, then a small tie strength value 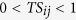 is assigned indicating a weak tie between the two players of different types.

Classical prisoner’s dilemma game model assumes that a cooperator invests *c* equally to each of the neighbors and the recipient gets *b* out of the investment *c*. In this model, as we introduced above, the investment preference of each player depends on his/her behavioral pattern. The investment from the prosocialist *i* to its opponent *j* (*I*_*ij*_) in each round is proportional to the tie strength between them, i.e. *i* invest 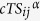 to *j* and *j* gets 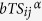. Similarly, if *i* is antisocial, then 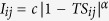. Egoists never invest to anyone, and altruists invest *c* to every neighbor regardless of the tie strength (See below the payoff matrix). *α* in the model is a tunable parameter controlling the strength of the investment preference. Prosocialists(antisocialists) donate more(less) to the intimate friends if *α* > 0. When *α* > 0 the model becomes a classical model containing only egoists(All-C) and altruists(All-D).

In each round, players interact with all the neighbors to accumulate their payoff. Random sequential update rule is applied in the model. Player *x* is allowed to adopt the strategy of a randomly selected friend *y* with a probability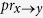 proportional to their payoff difference:


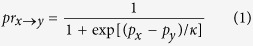


where *κ* is a parameter characterizing bounded rationality during evolution^10^, *κ* = 0 represents complete rationality, 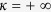 represents complete randomness. We set *κ* = 0.1 in this study.

## Simulation Results and Discussions

Before the game starts, equal percentage of the four types of players is randomly distributed among the whole population and the strength of each tie *TS*_*ij*_ is randomly drawn from the interval [0,2] indicating a random relationship at the initial state. The spatial prisoner’s dilemma game is iterated forward in time using a random sequential update scheme and all players are permitted to interact with their four nearest neighbors. The simulations are performed on a 100 × 100 square lattice, and the equilibrium fraction of cooperators is obtained by averaging over 1000 generations after a transient time of 10^7^ generations. The figures showing values of player densities on the spatial grid resulted from an average over 100 simulations with different distributions of initial tie strengths and players of different behavioral patterns.

**Figure 1:**
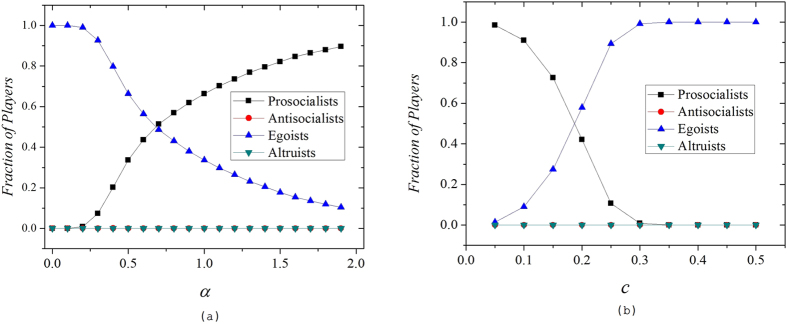
(**a**) The fraction of different types of players as a function of investment preference *α*. We set *b* = 1.1, *c* = 0.1 here. The figure shows that the introduction of *α* inhibits egoists and promotes prosocialists. Extremely strong *α* leads to global relationship-based cooperation. Antisocialists and altruists are always suppressed in the model regardless of *α* value. **(b)** The fraction of different types of players as a function of cost *c*. We set *α* = 2, *b* = 1 + *c* here in the model. As we expected, increasing the cost to cooperate suppresses all types of cooperation. Only egoists benefit from high cooperation cost.

The figure clearly shows the fact that the emergence of prosocialists depends on the investment preference *α* brought by tie strength (see Fig. 1). Under the proposed mechanism, each type of the players will form small clusters with strong internal tie strength and these clusters connect with other clusters of different types via external weak ties. When *α* > 0, prosocialists contribute significant more investment to players within the cluster while invest little to those outside. In contrary, antisocialists donate more to players outside the cluster and pay little to players of the same type. As a result, the average payoff of prosocial clusters is significantly promoted by both the strong investment preference from the internal prosocialists and the external antisocialists. In reverse, the payoff of antisocial clusters will be dramatically undermined by the same mechanism. Therefore, prosocial clusters outperform any other opponents if *α* is positive and increasing *α* can further strengthen this effect. As we can see from the figure, prosocialists dominate the network when *α* > 2. The existence of tie strength allows prosocialists to firmly support each other as well as taking the advantage of antisocialists and altruists. In this way, the compact prosocial clusters are strengthened and expanded.

**Figure 2:**
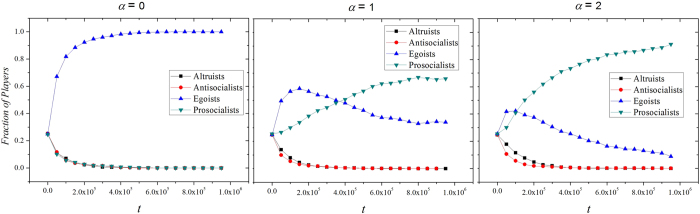
The evolution of four types of players with different *α*. We set *b* = 1.1, *c* = 1.1 here. When *α* > 0, the fraction of egoists exhibits a first up and then down pattern. This result indicates that isolated egoists win most 1 on 1 competitions and form large clusters initially. However, these non-cooperative clusters are gradually invaded by prosocialists if a moderate level of *α* is introduced.

**Figure 3:**
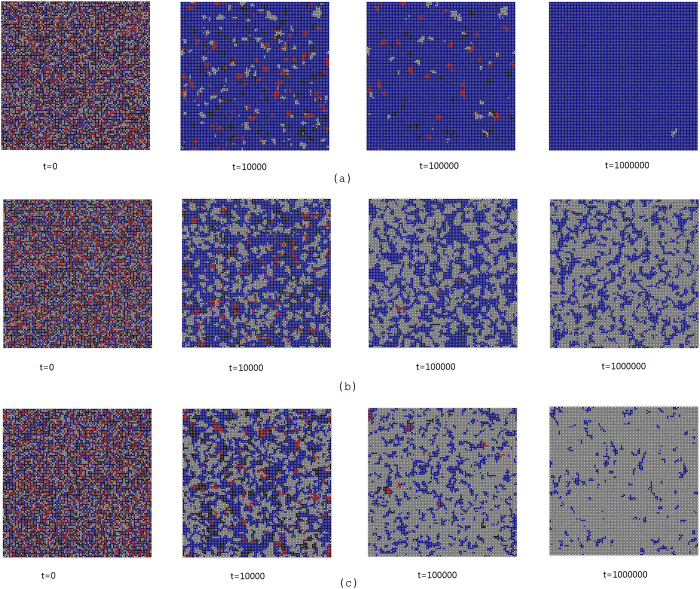
Snapshots of the evolutionary process for (a) 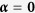 (b) 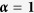 (c)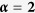. Figure 3 Snapshots of the evolutionary processes for different *α* on a 100 × 100 lattice. We set *b* = 1.1, *c* = 0.1 here. White nodes represents prosocialists, red for antisocialists, blue for egoists and black for altruists. The whole process can be summarized as two stages: cluster formation and cluster competition. First, the four types of players form clusters respectively. Second, clusters of different types compete for a living. It can be clearly observed from the figure that egoists dominate the lattice network when *α* = 0. However, prosocialists outperforms any other types if a significant level of relationship-based investment preference is introduced.

The above arguments can also be validated by the evolutionary process of different types of players under different *α* (see Fig. 2). When *α* is significant, we observe a turning point for the fraction of egoists. This pattern indicates that fact that the introduction of strong *α* will facilitate egoists to form clusters initially (see Fig. 3), isolated prosocialists, altruists and antisocialists are mostly likely to be converted into egoists. However, since egoist clusters produce lowest benefit while *α* brings additional benefits to prosocialists clusters, egoists will be gradually invaded by prosocialists. This effect leads to the final dominance of prosocialists in the evolution (see Fig. 3).

**Figure 4 f4:**
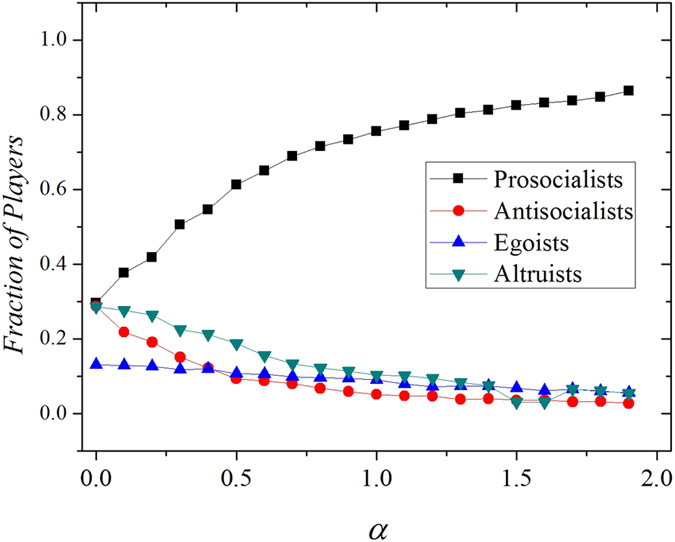
The fraction of different types of players as a function of investment preference *α* under DB updating rules. Strong *α* promotes prosocialists in our model.

We further explore the robustness of this conclusion by first adopting a death-birth (DB) updating mechanism. We use tag-based cooperation framework here^29^,^30^,^31^ to perform the simulation on a 100 × 100 empty lattice with periodic conditions, where the evolutionary process is defined as the following four stages: immigration, interaction, reproduction, and death. First, an immigrant with a random behavioral pattern enters the network and locates on a random empty site. Second, each immigrant in the lattice has its potential to reproduce (PTR) rate set to 0.12 initially, and agents plays with all its neighbors under our proposed interaction framework (see Table 2) to accumulate its PTR. E.g. a prosocialist *I* provides 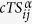 of its PTR to *j* and the recipient *j* increases its PTR by 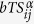 (we set *c* = 0.01, *b* = 0.03 in the simulation). Third, each agent has a chance to reproduce on a randomly chosen empty neighbor, and this probability is equal to its PTR. The offspring inherits the behavioral pattern of its parent with a mutation rate of 0.005, and a strong tie strength value 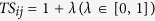 will be assigned to the tie between the parent and the offspring. Finally, each player has a 0.1 probability to die (Fig. 4)

The simulation results show that the above conclusion is also valid under DB updating mechanisms (see figure 4). Since imitation update rules explain the formation of individual behaviors and DB update rules model the evolution of biological species, the proposed relationship-based mechanism can be applied universally to interpret the emergence of cooperation(or cooperative behaviors) between individuals with strong ties. It is worth mentioning that the tag-based cooperation model can be regarded as a binary version of our tie strength model. In tag models, players are either friends or strangers, corresponding to either 1 or 0 tie strength values. Since various relationships exist even within the players of the same group, neglecting such heterogeneity will undoubtedly hinders our perception on players’ behaviors in games. This model not only explains why cooperation emerges between close friends(or “ethnocentrism” in tag models), but also reveals the reason why the more cohesive groups or organizations outperform others. The relationship based model provides a broader perspective for us to understand the emergence of cooperation in reality. [Fig f4]

**Figure 5:**
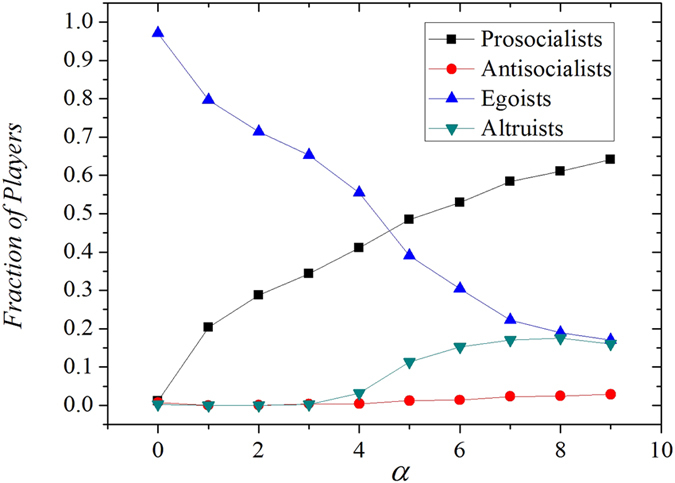
The fraction of different types of players as a function of investment preference. The degree-normalized payoff mechanism is applied. We set b = 1.1, c = 0.1 here. The simulations are performed on a square lattice, and the equilibrium fraction of cooperators is obtained by averaging over 1000 generations after a transient time of 1,000,000 generations. The figures showing values of player densities resulted from an average over 100 simulations with different distributions of initial tie strengths and players of different behavioral patterns.

Moreover, we test the validity of the model in different network structures. We perform the simulation test on a Barabasi-Albert (BA) network with the average connectivity of each node equals four. Since the lattice network represents homogeneous interaction networks, the structures of scale free networks are highly heterogeneous^32^,^33^,^34^. First, we have to emphasize that network reciprocity has already been proven to be dramatically promoted by using absolute payoffs on scale free networks^35^,^36^,^37^. It becomes difficult to observe the role of tie strengths in enhancing cooperation in a already cooperation-dominated environment. Second, the application of cumulative, absolute payoffs on strong heterogeneous networks already raised questions by other researchers, the most significant one of which is that it is usually expensive to maintain a large number of connections. Therefore, following Szolnoki *et al.*’s work^38^, we perform the simulation test on the BA network by coupling a degree-normalized payoff mechanism. The results presented in Fig. 5 also confirmed the previous argument that the relationship-based cooperation (prosocialists) is enhanced by the introduction of *α*. This conclusion is robust against the variation of network structures as long as the degree-normalized payoffs are applied.

## Summary

In previous sections, we introduce a tie strength model to explore the evolution of relationship-based cooperation under a game-theoretic framework. We show that the emergence of relationship-based cooperation (prosocialists) can be dramatically enhanced by coupling two simple mechanisms. The first mechanism presumes a tie strength-based investment preference, where players tend to investment more to the intimate friends. The second is the homophily or “birds of a feather” assumption, where tie strengths between similar individuals are assumed to be stronger than dissimilar ones. Considering four types of players with distinct behavioral patterns (prosocialists, antisocialists, egoists and altruists), the model reveals that the introduction of *α* always promotes relationship-based cooperation(prosocialists) regardless of the strategy-updating rules or the structural patterns of the networks. By observing the evolutionary process of the four types of players, we conclude two major stages of the interaction: cluster formation stage and cluster competition stage. First, the homophily assumption enables the four types of players form clusters with strong internal ties initially. Second, clusters compete with each others for evolutionary advantages. The simulation results show that egoists dominate the competition if no investment preference is included, while strong *α* strengthens the average payoffs of prosocial clusters to survive in the evolution.

The findings of the paper also have strong practical implications. As Harrison *et al.* discovered in their empirical experiment, it is human nature to suffer more costs to the intimate friends. Our models show that this born preference enables prosocialists outperform other strategists in the competiton. Moreover, by adopting a DB updating rule, we show that this tie strength model can be regarded as a continuous version of tag-based cooperation model discussed extensively in the subject^29^,^30^,^31^. In tag models, the relationship of the two players are actually defined as either friends (tie strength value equals 1) or strangers (tie strength value equals 0). Therefore, it fails to explain how continuous tie strength distributions and the subsequent investment preferences affect the interactions among players. Our proposed model not only unveils the underlying motivation that leads to the emergence of relationship-based cooperation (or “ethnocentrism” in tag models), but also reveals that the level of prosocialists can be further enhanced by strengthening the internal bonds within the prosocial clusters. This conclusion interprets the phenomenon mentioned in the introduction section why groups with strong internal bonds outperform other groups in reality. This research may provide a brand new perspective to understand the evolution of relationship-based cooperation.

## Additional Information

**How to cite this article**: Xu, B. and Wang, J. The Emergence of Relationship-based Cooperation. *Sci. Rep.* **5**, 16447; doi: 10.1038/srep16447 (2015).

## Figures and Tables

**Table 1:** The payoff matrix of classical donation game.

	C	D
C	*b* – *c*, *b* – *c*	*− c, b*
D	*b − c*	0, 0

**Table 2:** The payoff matrix of the relationship-based model.

	Prosocialist	Antisocialist	Egoist	Altruist
Prosocialist	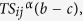	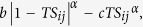	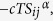	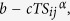
	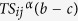	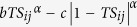	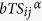	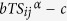
Antisocialist	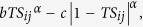	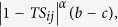	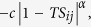	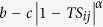
	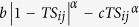	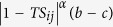	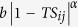	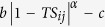
Egoist	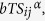	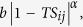		
	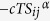	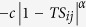	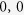	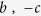
Altruist	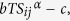	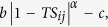		
	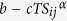	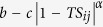	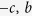	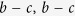
